# Pulsed Corona Discharge Induced Hydroxyl Radical Transfer Through the Gas-Liquid Interface

**DOI:** 10.1038/s41598-017-16333-1

**Published:** 2017-11-23

**Authors:** Petri Ajo, Iakov Kornev, Sergei Preis

**Affiliations:** 1 School of Engineering Science, Lappeenranta University of Technology, P.O. Box 20, 53851 Lappeenranta, Finland; 2 School of Advanced Manufacturing Technologies, Tomsk Polytechnic University, 2A Lenina Ave., 634028 Tomsk, Russia

## Abstract

The highly energetic electrons in non-thermal plasma generated by gas phase pulsed corona discharge (PCD) produce hydroxyl (OH) radicals via collision reactions with water molecules. Previous work has established that OH radicals are formed at the plasma-liquid interface, making it an important location for the oxidation of aqueous pollutants. Here, by contacting water as aerosol with PCD plasma, it is shown that OH radicals are produced on the gas side of the interface, and not in the liquid phase. It is also demonstrated that the gas-liquid interfacial boundary poses a barrier for the OH radicals, one they need to cross for reactive affinity with dissolved components, and that this process requires a gaseous atomic H scavenger. For gaseous oxidation, a scavenger, oxygen in common cases, is an advantage but not a requirement. OH radical efficiency in liquid phase reactions is strongly temperature dependent as radical termination reaction rates increase with temperature.

## Introduction

Atmospheric pressure non-thermal plasma technologies have been an on-going focus of research in water treatment over the last two decades. The most common laboratory-scale studies have included investigation of dielectric barrier discharge (DBD) and pulsed corona discharge (PCD), the latter forming the subject of discussion in this paper. Plasma treatment of water is chemically similar with traditional ozonation, as several oxidants are formed in plasma reactions from oxygen and water, including ozone O_3_, hydroxyl radical OH, hydrogen peroxide H_2_O_2_, atomic oxygen O(^3^P) and singlet oxygen O_2_(^1^Δ_g_). Of these, O_3_ and OH are specially recognized as the major oxidants in plasma water treatment^1–5^. The latter is largely produced via inelastic electron collisions with water as in Eq. (1)^3^:

1 $${{\rm{H}}}_{2}{\rm{O}}+{e}^{-}\to {\rm{OH}}+{\rm{H}}+{e}^{-}$$

For water treatment purposes, the gas-liquid interface where the OH radicals are formed is a crucial environment and the plasma-liquid contact surface area thus makes an important variable^4–6^. In earlier work, a vertical PCD reactor in which the treated solution is allowed to shower through a perforated plate positioned above the plasma zone was employed to generate a large plasma-liquid contact surface area^6^. Increasing this contact surface improves the oxidation energy efficiency mainly due to the enhanced action of superficial OH radicals^4,6^. Although OH radical activity at the plasma-liquid interface is evident, it is extinguished in complete absence of O_2_^4^. So far this property, however, seems only observed with the present configuration; several other types of non-thermal plasmas applied in oxidation of aqueous organic compounds seem to have effect also in the absence of molecular O_2_^7–11^, suggesting that these processes include various mechanisms. The occurrence of such reports is understandably limited as in most water treatment studies the gas phase contains air for practical reasons, and thus O_2_ at least as a partial constituent. Oxidation for example under Ar has shown similar efficiency with air^11^, but papers describing oxidation under pure nitrogen plasma are scarce. More importantly, these reports tend to describe plasma discharge types that substantially differ from our PCD configuration.

It should be noted that here, the absence of O_2_ does not suggest that Eq. (1) would be invalid under these circumstances. Instead, it is proposed in^4^ that the atomic hydrogen H (a product in Eq. (1)) recombines with the OH radical in the absence of O_2_, which is a strong H scavenger following Eq. (2)^5^.

2 $${\rm{H}}+{{\rm{O}}}_{2}+M\to {{\rm{HO}}}_{2}+M$$

In other words, OH radicals are not able to react with the dissolved species if H is not scavenged. It is, however, interesting to consider why OH radicals in this case are completely unreactive in the liquid phase without O_2_, while they can be effective even in anaerobic natural waters^12^. In the present study, we address this problem by suggesting that OH radicals are formed only from water vapor and their transfer through the gas-liquid interface is the decisive process for successful oxidation of dissolved components. We arrive at this conclusion by describing: (i) OH radical oxidation of gaseous nitrogen and acetone without ambient O_2_; (ii) zero oxidation of oxalic acid (OA) in the liquid phase in the absence of O_2_; and (iii) demonstrating the temperature dependence of the possibility of the OH radical diffusing unreacted through the interface under air plasma. These observations provide essential information for the conceptualization of new PCD-based OH radical processes by enabling mapping of the kind of redox reactions that are achievable.

## Results and Discussion

Ambient plasma is known to result in oxidation of N_2_, producing dissolved NO_x_ in water treatment applications. In this study, the experiments showed temperature and pulse frequency as having no effect on the yield of NO_x_ (per energy dose), measured as aqueous total nitrogen (TN). Since water vapor pressure increases with temperature, it would be reasonable to assume that the density of gaseous OH radicals would correlate with it. The observed temperature independence of N_2_ oxidation is therefore very interesting, as it does not display any correlation to water vapor pressure, which at 13…30 °C ranges from 1.51 to 4.25 kPa.

**Figure 1:**
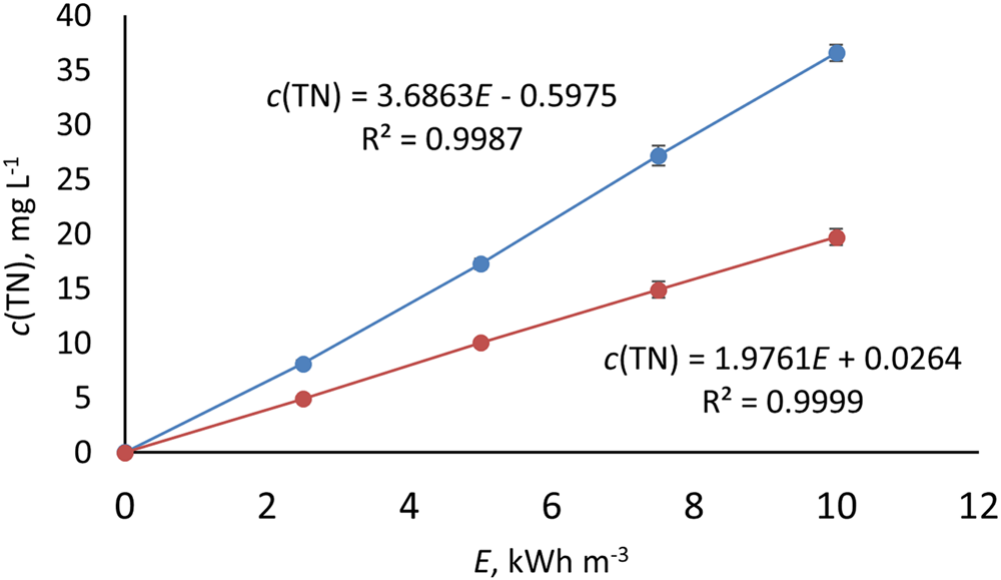
Average dissolved TN evolution in air (blue) and nitrogen (red) atmosphere during PCD oxidation. The experiments were conducted at 13, 20 and 30 °C and at pulse frequencies of 300, 500 and 833 pps. *E* = specific plasma energy dose.

In agreement with previous studies^13,14^, the indifference towards pulse frequency indicates that only short-lived species present during and shortly after the pulses contributed to NO_x_ formation (OH radical lifetime on water surface is ~2.7 µs^15^, 1–2 orders of magnitude longer above the surface^5^). Under N_2_, however, the TN formation rate at 1.98 g kWh^−1^ was almost half that in air, 3.69 g kWh^−1^. It should be noted that the yield in air is over twice the TN 1.77 g kWh^−1^ calculated from NO_3_^−^ formation presented in^14^, which is probably due to the water being introduced in aerosol form, rather than the previously applied showering approach, substantially increasing the gas-liquid contact area (see Experimental Methods). The dissolved TN evolution observed under N_2_, i.e. in the absence of O_2_, suggests OH radical induced oxidation taking place in the gas phase because simultaneously there was zero oxidation in the liquid phase, as will be discussed below. The difference in formation rates under air and N_2_ indicates that nitrogen oxidation is supported by oxygen, which acts as a strong atomic H scavenger and contributes to the formation of strong oxidants. The evolution of TN concentration under air and N_2_ is presented in Fig. 1. Since variation in the process parameters (temperature and pulse frequency) had no effect on TN formation rate against delivered energy, the values presented are averages of all experiments under a given atmosphere.

In agreement with previous findings^14^, the dissolved TN in treatment under air was found to consist only of NO_3_^−^. Without oxygen, also NO_2_^−^ was identified, which may simply be explained by the absence of O_3_ since dissolved O_3_ readily oxidizes NO_2_^−^, producing mainly NO_3_^−^ and O_2_(^1^Δ_g_)^16^.

**Figure 2:**
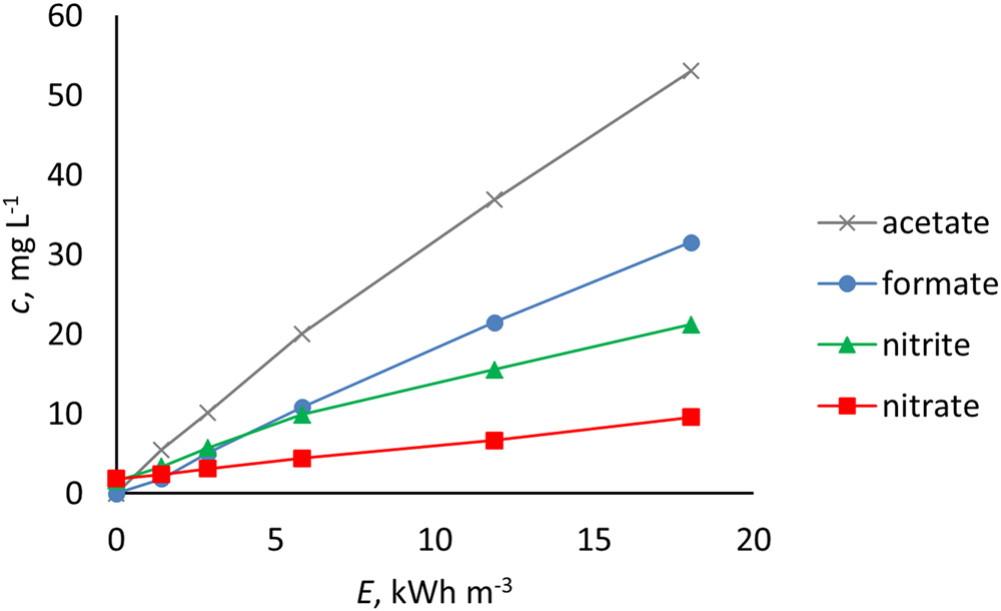
Oxidation product evolution in oxidation of 17% aqueous acetone under N_2_.

The gas phase oxidation was confirmed with concentrated acetone solutions (17%); acetone oxidation under N_2_ resulted in notable accumulation of dissolved oxidation products, acetic^17^ and formic acid^18^, during the treatment. The IC detector response for acetate and formate showed an increase consistent with delivered energy dose. Some inaccuracy of the acetate and formate concentrations may occur due to the partial overlapping of the chromatogram peaks (the chromatograms are presented in Supplementary Discussion 1). The evolution of the oxidation products, however, confirm the gas phase formation of OH radicals, whereas with the dissolved probe compound (OA), there was no indication of liquid phase OH radical activity under N_2_ atmosphere (discussed further below). It should be noted that under these conditions, the presence of lower oxidation state NO_x_ may enhance the OH induced oxidation process via secondary reactions^19^, the extent of which may provide excellent topics for further research. The ion chromatograms of the acetone oxidation products with NO_2_^−^ and NO_3_^−^ are presented in Fig. 2.

**Figure 3:**
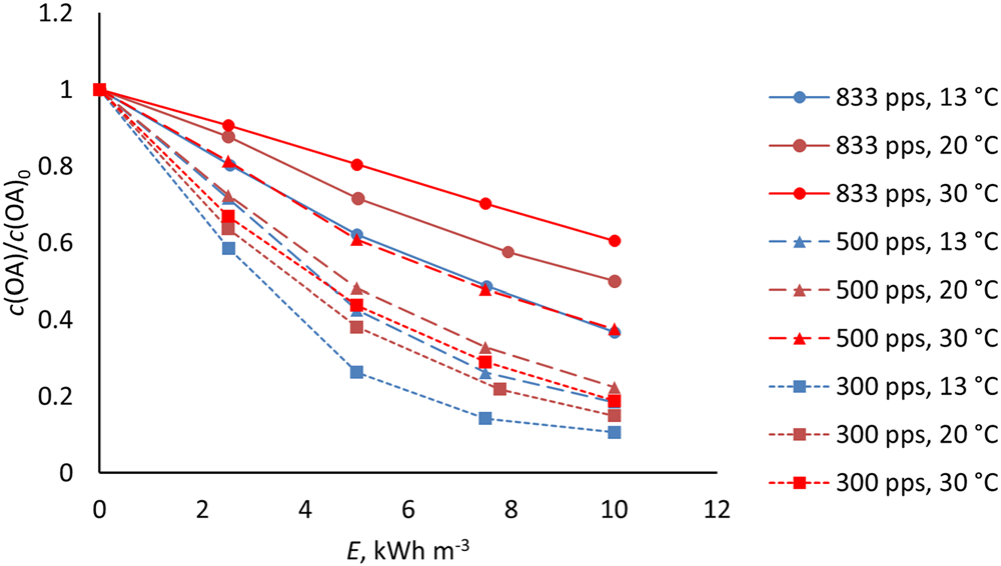
Oxalic acid oxidation at different temperatures and PCD pulse frequencies from initial concentration of 60 mg L^−1^. *E* = specific plasma energy dose.

Temperature and pulse frequency displayed consistent correlation with OA oxidation energy efficiency in the experiments conducted under air, the reaction rates exhibiting kinetic profile change from zero to first order along the decrease in both parameters from the highest to the lowest applied values, as can be seen in Fig. 3.

The oxidation efficiency at 300 and 500 pps is at similar range while distinctly lower at 833 pps. The difference stems mainly from the role of O_3_^6^. At equal energy doses, the amount of pulses and thus the amount of OH from Eq. (1) can be considered constant: the role of O_3_ can then be considered from the difference in treatment times at different frequencies. The OA oxidation yield at 833 pps is almost halved from 13 to 30 °C, while its improvement when changing to 500 pps, attributable to longer treatment time, varies very little at any temperature, which emphasizes the temperature dependence of OH radical in the oxidation of the dissolved species (calculations in Supplementary Discussion 2).

**Figure 4:**
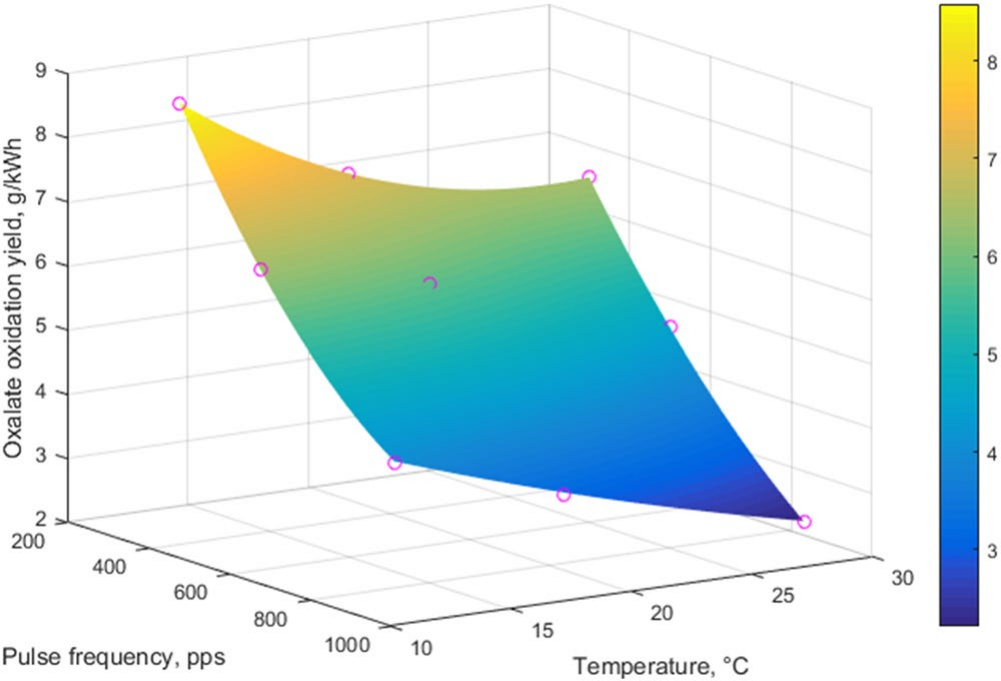
Oxalic acid reaction yield dependence on temperature and pulse frequency (zero order reaction 0…5 kWh m^−3^).

By taking the reaction rates from the 0…5 kWh m^−3^, wherein the degradation is practically linear in all cases (see Fig. 3), the frequency dependent decelerations of the OA oxidation rates caused by temperature increase can be directly visualized (Fig. 4).

Given the very low reactivity of O_3_ and OA at low pH, the magnitude of the temperature effect seen in Fig. 4 - strong even at high pulse frequency - is largely attributed to OH radicals. For reference, at similar concentrations and pH, aqueous OA reduction by O_3_ alone was reported in^20,21^ as a maximum of ~10% for 30…60 min treatment. The applied O_3_ concentration ranged from similar^20^ to an order of magnitude higher^21^ compared to our process^7^. Since formation of OH radicals is induced by electrons with ~3 orders of magnitude higher temperature than that of ions and molecules^22^, the rate of it cannot practically be dependent on the gas temperature range in the current experiments. It can be therefore concluded that high temperature increases OH radical activity instead, which, however, results in decreasing the oxidation rates of dissolved components due to promotion of premature radical termination reactions. Several possibilities for these reactions are described in^23,24^.

Under N_2_, TOC remained unchanged i.e. zero oxidation for OA was observed at any combination of pulse frequency and applied temperature. The result is in agreement with earlier research, where even phenol remained unoxidized in PCD treatment without molecular oxygen^4^. The liquid phase OH radical formation testing by using KMnO_4_ as an atomic H scavenger also yielded zero oxidation of OA, further demonstrating that no OH radicals are formed in the liquid phase, and that the radicals must therefore come from the gas side of the interface. During the treatment, the permanganate was gradually reduced to Mn(IV)O_2_, which is an inevitable development due to the NO_x_ formation. This development is presented in Supplementary Discussion 3. These findings extend and are supported by the report of Kanazawa *et al*.^5^, where gas-phase OH radical formation and its dissolution into liquid phase are discussed and OH lifetime on water surface found to be substantially shorter than in humid air.

In summary, via simple experiments with a PCD plasma water treatment system under air and N_2_, we have showed that although OH radicals are active also in the liquid phase, in the present kind of plasma-water interaction they are formed in the gas phase only (i.e. from water vapor), and that the gas-liquid interface is a major barrier for the efficient utilization of the radicals in oxidation of aqueous compounds. The absence of atomic H scavengers in the gas phase promotes OH recombination with H, which is too fast for OH radicals to cross the gas-liquid interface boundary unreacted. Liquid-phase H scavenging does not enable OH induced oxidation, suggesting that no radicals are produced on the liquid side. In the absence of gaseous H scavengers, oxidation by OH still occurs in the gas phase because the radicals do not need to cross an interfacial boundary. Temperature increases OH radical reactivity, which hinders oxidation energy efficiency of dissolved compounds by promoting premature reactions, i.e. reactions occurring prior to successful transfer through the interface.

## Methods

**Figure 5:**
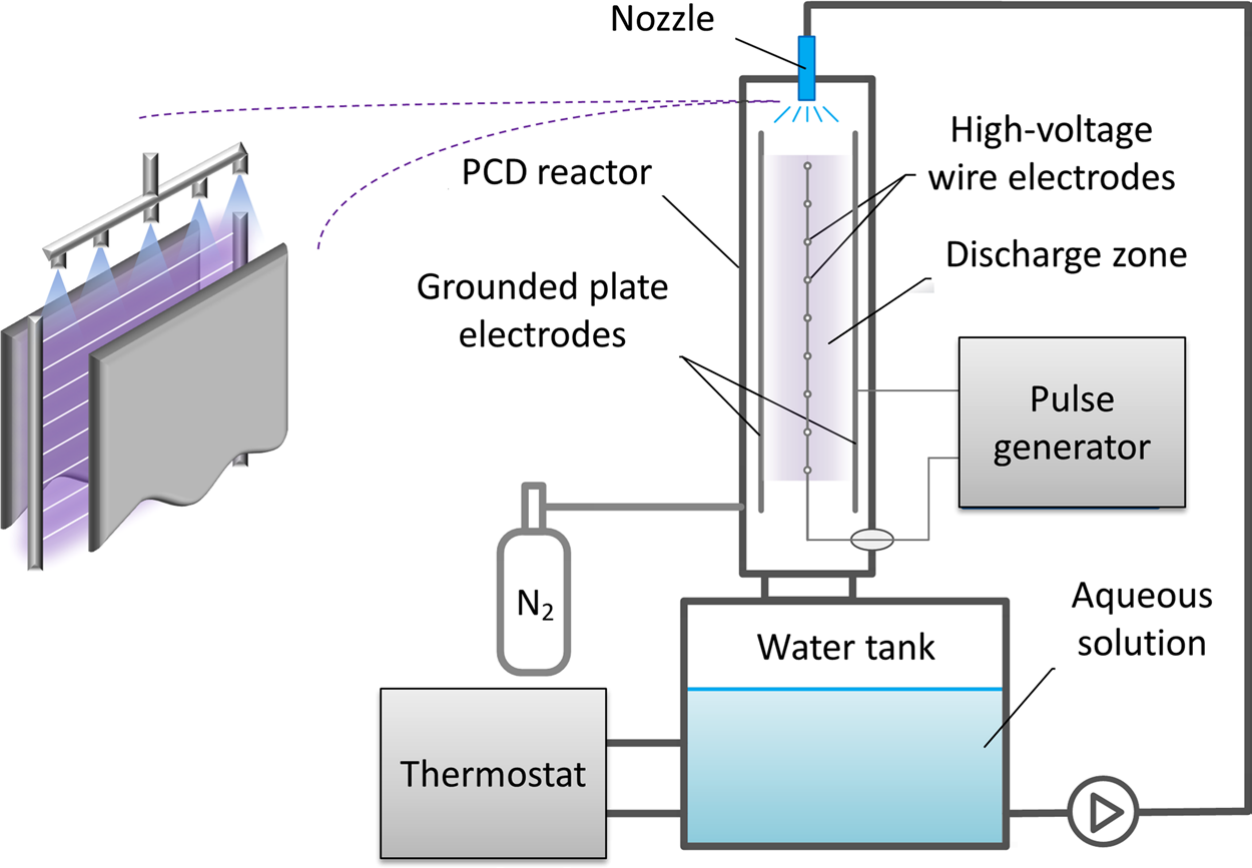
Top section of the PCD reactor with the atomizer array spraying the water into the plasma zone and a schematic illustration of the experimental configuration. The latter depicts a reactor view parallel to the electrodes.

The PCD reactor used is a vertical wire-plate configuration with a pulse generator adjustable to deliver identical pulses (22 kV and 180 A peak amplitude) at 50 to 833 pulses per second (pps) at corresponding nominal power of 6 to 100 W (0.12 J per pulse). Water was sprayed from above into direct contact with the plasma, using five axial-flow full cone atomizer nozzles, at volumetric flow rate of 1.8 L min^−1^. Below the reactor is a tank holding the treated solution, a jacketed vessel coupled with a thermostat for adjusting the operating temperature. For experiments under N_2_, oxygen absence was confirmed by measuring gas composition in the water tank headspace, using an oxygen analyzer (based on paramagnetic susceptibility measurements). The practical concept is illustrated in Fig. 5. A more detailed technical description of the PCD system (excluding the atomizer setup) and a pulse oscillogram can be found in our previous publication^6^.

Oxalic acid (OA) concentrations were measured by analyzing the total organic carbon (TOC) concentrations (applying catalytic combustion at 680 °C) coupled with a total nitrogen (TN) unit for simultaneous TN analysis. Since OA has no organic oxidation products, i.e. is directly oxidized to CO_2_, TOC corresponds directly to OA concentration. Similarly, in the experiments under air, TN value can be used to determine NO_3_ concentrations since PCD yields no other dissolved NO_x_ species under air, as mentioned above. Acetone oxidation products, formate and acetate, were observed using an ion chromatograph (IC) with an anion column. In the experiments under N_2_ atmosphere, IC was also used to identify any presence of NO_2_^−^.

For the OH reactions with dissolved components, OA at 60 mg L^−1^ (pH ~3.4) was used as the organic probe compound for favoring OH radical over O_3_ reactions, following the low reactivity of OA with ozone (*k* ≤ 4 ∙ 10^−2^ M^−1^ s^−1^ ^25^ at pH 5–6; for the reaction with OH *k* = 5.3 ∙ 10^6^ M^−1^ s^−1^ ^26^), emphasized under acidic conditions. The experiments were conducted in 10 L batches and sampling was done at 2.5 kWh m^−3^ intervals of discharge energy per treated water volume until 10 kWh m^−3^. The experiments were carried out at three pulse frequencies, 300, 500 and 833 pps: due to the identic pulses, increasing frequency at fixed energy doses results in reduced treatment time, lower frequency therefore giving the longer living species, like O_3_, more time to react with the probe compound. The experiments were conducted at temperatures of 13, 20 and 30 °C.

For the experiments under N_2_, the system was flushed with N_2_ until all oxygen was replaced. For these experiments, the treated solution was also degassed before introduction into the tank and kept running through the system under N_2_ atmosphere for 40 minutes before starting the experiment to ensure negligible dissolved oxygen. N_2_ inflow was kept at 7 L min^−1^ throughout the experiments to ensure zero oxygen intake from ambient air. To ascertain whether OH radical formation in the liquid phase occurs, potassium permanganate KMn(VII)O_4_ (0.1 mM) was used in a separate experiment as an atomic H scavenger in the dissolved phase. For any reaction following Eq. (1), the permanganate would not be oxidized by OH but instead serve as an oxidant for atomic H. Under N_2_, thus scavenged atomic H in the liquid phase would enable OH radical reactions with OA. To exclude oxalate oxidation by permanganate, sodium oxalate was in this case used at neutral pH and at the lowest applied temperature of 13 °C, under which conditions permanganate is not reactive with oxalate.

Gas phase oxidation under N_2_ was studied with a 17% acetone solution, exploiting the high volatility of the substance. Run under N_2_ atmosphere, acetone was used to demonstrate organic species oxidation in the gas phase by OH radicals from water vapor, in the absence of O_2_. The experimenting was conducted at 833 pps and 20 °C and acetone solution volume was reduced to 7 L for convenience, and the process overall duration was extended to 18.03 kWh m^−3^ of delivered energy to highlight the observable effects.

### Data Availability

The datasets generated during the current study are available from the corresponding author on reasonable request.

## Electronic supplementary material

Supplementary Information
