# Supplementary material for: Pulsed Corona Discharge Induced Hydroxyl Radical Transfer Through the Gas-Liquid Interface
Source: Sci Rep. 2017 Nov 23;7:16152. doi: 10.1038/s41598-017-16333-1 (PMC5700971; doi:10.1038/s41598-017-16333-1)
Supplement: Supplementary file 1 — Supplementary Information [file 41598_2017_16333_MOESM1_ESM.doc]

## Pulsed Corona Discharge Induced Hydroxyl Radical Transfer Through the Gas-Liquid Interface

*PETRI AJO^a^, IAKOV KORNEV^b^, SERGEI PREIS*^c^*

**Supplementary Discussion 1.** Ion chromatograms of the acetone oxidation samples.

The ion chromatograms are presented in Fig. S1. Acetone (3.70 min) and formate (3.90 min) peaks overlap, which causes some uncertainty in the accuracy of the concentrations. Regardless, however, the consistent increase in the oxidation products demonstrate the gaseous oxidation occurring under N_2_, when none is observed in the liquid phase.


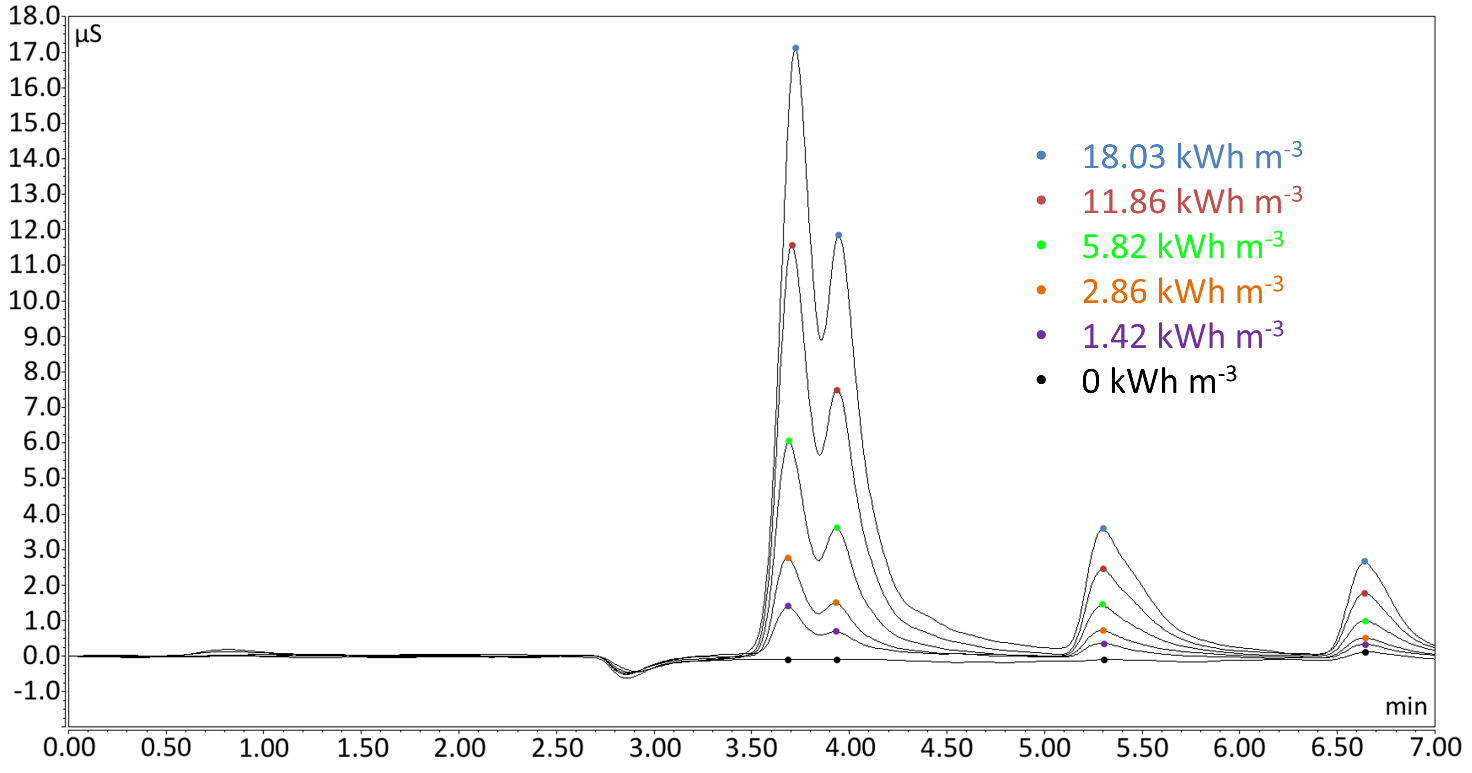


**Figure S1.** Ion chromatograms for acetone oxidation under N_2_: the double peak corresponds to acetate (3.70 min) and formate (3.90 min), followed by nitrite (5.30 min) and nitrate (6.70 min).

**Supplementary Discussion 2.** Calculation of the difference in OA oxidation yield at 833 and 500 pps pulse frequencies over the studied temperature range.

Table S1 below demonstrates the calculation of yield improvement (g kWh^-1^) over treatment time extension (g kW^-1^h^-2^) at frequency change from 833 to 500 pps (*y* = yield, *t* = treatment time). The data is taken from treatment until 5 kWh m^-3^, during which the oxalic acid (OA) degradation was practically linear. Only these two frequencies were included in the calculation as the different roles of ozone and OH radical are more observable at higher frequency, as indicated in the text.

**Table S1.** Change in oxalic acid oxidation yield (*y* = yield, *t* = treatment time)

| *f* | *833 pps* | *500 pps* |  |  |
| --- | --- | --- | --- | --- |
| *P* | 100 W | 60 W | *t*_500_-*t*_833_ |  |
| *t* | 0.485 h | 0.808 h | 0.323 h  *y*_500_-*y*_833_, g kWh^-1^ |  |
|  | *y*, g kWh^-1^ | |  |
| 13 °C | 4.06 | 6.38 | 2.32 |  |
| 20 °C | 3.18 | 5.79 | 2.61 |  |
| 30 °C | 2.20 | 4.55 | 2.36 |  |

The yield at 833 pps nearly halves from 13 to 30 °C (4.06 to 2.20 g kWh^-1^), while the improvement from decreasing frequency (ranging 2.32…2.61 g kWh^-1^) seems little affected by the operating temperature. In other words, slowing down the frequency, which gives longer living species like O_3_ more time to react, produces similar improvement at each temperature suggesting that here the longer living species play minor role and the temperature effect is mainly observed on OH radicals.

**Supplementary Discussion 3.** Reduction of aqueous permanganate to manganese dioxide in PCD under N_2_.

The presence of any NO_x_ species at lower oxidation state than NO_3_^-^ provides reactants for the permanganate, which is seen as the reduction of the permanganate to form insoluble MnO_2_. Figure S2 presents this reduction during the process, i.e. along increasing NO_x_ concentration. No reaction between oxalate and permanganate was observed. Although the permanganate disappears during the process, it should be noted that oxalate remained unoxidized at any permanganate concentration, indicating that no atomic H is present to be scavenged and no OH thus formed in the liquid phase.


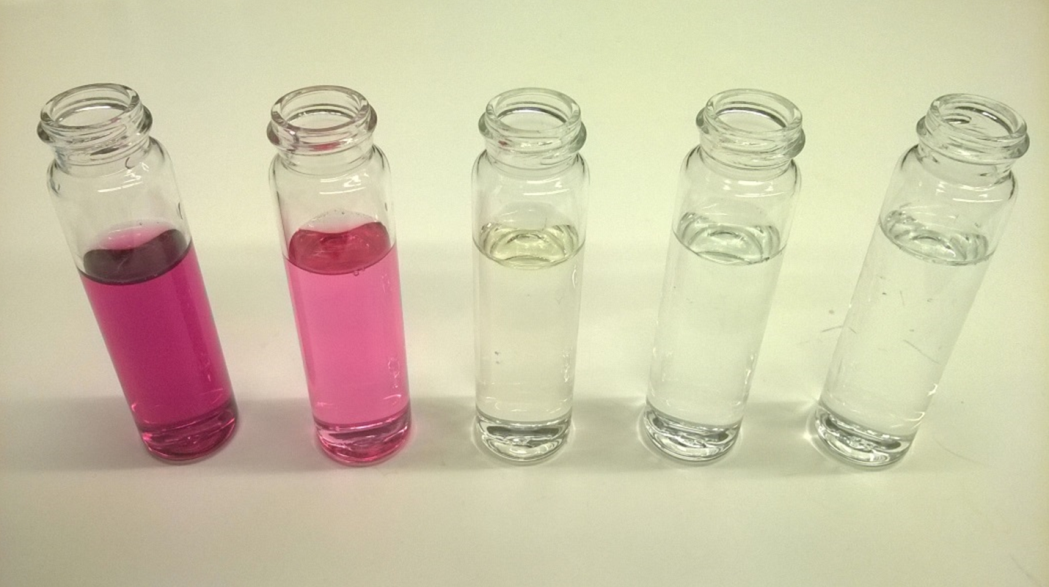


**Figure S2.** Filtered samples of permanganate solution treated with PCD under N_2_. From left to right: 0; 2.5; 5.0; 7.5; 10 kWh m^-3^. MnO_2_ was removed from the samples with a 0.45 µm syringe filter.
